# Optimized Crop Disease Identification in Bangladesh: A Deep Learning and SVM Hybrid Model for Rice, Potato, and Corn

**DOI:** 10.3390/jimaging10080183

**Published:** 2024-07-30

**Authors:** Shohag Barman, Fahmid Al Farid, Jaohar Raihan, Niaz Ashraf Khan, Md. Ferdous Bin Hafiz, Aditi Bhattacharya, Zaeed Mahmud, Sadia Afrin Ridita, Md Tanjil Sarker, Hezerul Abdul Karim, Sarina Mansor

**Affiliations:** 1 Department of Computer Science & Engineering, Bangabandhu Sheikh Mujibur Rahman Science and Technology University, Pirojpur 8500, Bangladesh; shohag3340csecu@gmail.com; 2 Faculty of Engineering, Multimedia University, Cyberjaya 63100, Malaysia; tanjilbu@gmail.com (M.T.S.); hezerul@mmu.edu.my (H.A.K.); 3 Department of Computer Science & Engineering, University of Liberal Arts Bangladesh, Dhaka 1207, Bangladesh; jaoherraihan@gmail.com (J.R.); niaz.ashraf@ulab.edu.bd (N.A.K.); ferdous.hafiz@ulab.edu.bd (M.F.B.H.); aditibhattacharya99@gmail.com (A.B.); zaeedmahmud@gmail.com (Z.M.); sadiaafrinhridita@gmail.com (S.A.R.)

**Keywords:** support vector machines (SVMs), convolutional neural networks (CNNs), classification, feature extraction, EfficientNetB0

## Abstract

Agriculture plays a vital role in Bangladesh’s economy. It is essential to ensure the proper growth and health of crops for the development of the agricultural sector. In the context of Bangladesh, crop diseases pose a significant threat to agricultural output and, consequently, food security. This necessitates the timely and precise identification of such diseases to ensure the sustainability of food production. This study focuses on building a hybrid deep learning model for the identification of three specific diseases affecting three major crops: late blight in potatoes, brown spot in rice, and common rust in corn. The proposed model leverages EfficientNetB0′s feature extraction capabilities, known for achieving rapid high learning rates, coupled with the classification proficiency of SVMs, a well-established machine learning algorithm. This unified approach streamlines data processing and feature extraction, potentially improving model generalizability across diverse crops and diseases. It also aims to address the challenges of computational efficiency and accuracy that are often encountered in precision agriculture applications. The proposed hybrid model achieved 97.29% accuracy. A comparative analysis with other models, CNN, VGG16, ResNet50, Xception, Mobilenet V2, Autoencoders, Inception v3, and EfficientNetB0 each achieving an accuracy of 86.57%, 83.29%, 68.79%, 94.07%, 90.71%, 87.90%, 94.14%, and 96.14% respectively, demonstrated the superior performance of our proposed model.

## 1. Introduction

Agriculture has been a way of life since the beginning of human civilization. It progressed from traditional practices to modern technologies. However, over time, pests and crop diseases have evolved as well and it is crucial to detect crop disease manifestations in the crops as early as possible due to the rise of global food demand. Diseases impact both the quantity and serve as its main host; they can also affect various other plants, such as tomatoes and petunias, as well as the quality of crop production, food security, economic stability, and livelihoods. Late blight is caused by a fungus-like pathogen known as oomycete. While potatoes and hairy nightshades are susceptible to late blight, it is crucial to differentiate between the two for accurate disease management [1]. The initial symptoms are little, light to dark green, round to irregularly shaped, water-soaked patches on the leaves, and these lesions usually appear on the lower leaves. These lesions grow quickly into huge, dark brown or black lesions during cool and moist weather. The first epidemic of late blight happened over 150 years ago in Europe [2]. It is now seen as a common example of an obligate parasite that can affect a large number of crops. New infections occur and entire leaves can become blighted and killed within just a few days. Late blight can spread from these local sources to potato fields and there are high chances of crops being affected by late blight particularly in regions where certain areas remain damp for extended periods. Brown spot has been largely recognized as one of the most common and damaging rice diseases. The disease causes 5% yield loss across all lowland rice production in South and Southeast Asia on an average basis, and severely infected fields can have yield losses as high as 45% (IRRI, 2020). Infected parts have small, circular, yellow-brown, or brown lesions, and fully developed lesions are circular to oval with a light brown to gray center. It can grow well in relatively high humidity. Puccinia sorghi is the fungus that causes common rust, which happens every growing season. Dead tissue lesions may appear as the leaf tissue surrounding the pustules becomes yellow or dies. When a leaf is highly affected, the lesions can occasionally form a band across the leaf. Common rust can spread rapidly in warm and humid conditions [3].

The authors in [3] propose a model that combines convolutional neural networks (CNNs) and support vector machines (SVMs) for multi-classification based on the use of motor imagery techniques. Batch normalization and dropout layers were incorporated into CNN to improve overfitting. After carrying out a comparative study on performance matrices, the average cross-validation accuracy rates for CNN and CNN-SVM were 82% and 84.1%, respectively. The experimental results indicate that the CNN-SVM model performs better than CNN and many existing classification algorithms. In 2021, Bansal et al. [4] proposed an ensemble model of pre-trained DenseNet121, EfficientNetB7, and EfficientNetB0. It classified the leaves of apple trees into categories such as healthy, apple scab, apple cedar rust, and multiple diseases using its images. The authors included various image augmentation techniques to increase the dataset size and enhance the model’s accuracy. The proposed model achieved an accuracy of 96.25% on the validation dataset. A study conducted by Afzaal et al. [5] evaluated the potential of utilizing deep learning (DL) combined with machine vision to detect early blight disease in potato production systems. Three types of convolutional neural networks (CNNs)—GoogleNet, VGGNet, and EfficientNetB0—were trained using the PyTorch framework to analyze disease images at different growth stages. The images were categorized into three dimensions (2-class, 4-class, and 6-class). The outcome of this study revealed that EfficientNetB0 performed significantly better than other CNNs, achieving an FScore range of 0.79 to 0.94. In this study, traditional machine learning (ML) and CNN-based transfer learning approaches were presented for cucumber disease recognition. The performance of both techniques was compared to find the most appropriate one. Then, various ML algorithms were compared using k-means-based image segmentation after extracting 10 relevant features. Random forest achieved the better accuracy with 89.93%. In the case of CNN-based transfer learning, a comparison among various InceptionV3, MobileNetV2, and VGG16 was carried out, and MobileNetV2 achieved the highest accuracy with 93.23%. There were 4200 images after data augmentation, and this dataset was divided into train, validation, and test sets. A total of 20% data was kept for testing purposes; among the rest of the 80% data, 20% data was used for the validation of the models, and the remaining data was used for the training of the models [6]. In another study by F. T. Pinki et al. [7], an automated system was designed to diagnose three common diseases affecting paddy leaves: brown spot, leaf blast, and bacterial blight. The system provides recommendations for pesticides or fertilizers based on the severity of diseases. K-means clustering is applied to identify the affected areas in a paddy leaf image. Visual features such as color, texture, and shape are used as features for classifying these diseases using a support vector machine (SVM) classifier. After recognizing the disease, the system suggests a remedy. This study addresses the issue of sugarcane diseases, which can lead to significant financial losses for small scale farmers. The deep learning model used in this study was trained and tested on a dataset of 13,842 sugarcane images containing both healthy and disease-infected leaves, and it achieved an impressive accuracy of 95%. The authors, Militante et al. [8], employed CNN with seven different classes to classify the sugarcane leaf. Jadhav et al. [9] used pre-trained CNN models to identify diseases in soybean plants using a transfer learning approach. CNN models such as AlexNet and GoogleNet attained better outcomes, but the model lacked in the diversity classification. Rather than creating a model to classify different plant diseases, many of the existing models focus on identifying a single class of a plant disease. This is mainly due to the limited datasets to train deep-learning models for a diverse range of plants. The classification of plant diseases using the ensemble classifier was proposed in another study by Astani et al. [10]. The best ensemble classifier was evaluated with two different datasets. The study conducted by Kolluri et al. [11] uses multimodal learning on colored datasets and grayscale datasets. The models performed better on colored datasets rather than grayscale ones. In this research by Eunice et al. [12], (CNN)-based pre-trained models were utilized for efficient plant disease identification. The researchers focused on fine-tuning the hyperparameters of popular pre-trained models, such as DenseNet-121, ResNet-50, VGG-16, and Inception V4. DenseNet-121 outperformed the other models. Table 1 illustrates the utilization of various machine learning models as well as contemporary deep learning models for image classification by researchers. Among deep learning models, VGGNet and EfficientNet appear to be the most used ones in the research reviewed by the authors.

Despite significant advancements in crop disease identification using deep learning and machine learning algorithms, several research gaps remain. Current studies often focus on single crop diseases, limiting their generalizability across diverse crops. Additionally, many models face challenges with computational efficiency and scalability. These limitations hinder the widespread adoption of such models in resource-constrained agricultural environments and leads to the research question “Can a hybrid deep learning and SVM model achieve high classification accuracy for multiple crop diseases affecting major crops in Bangladesh (potato late blight, rice brown spot, and corn common rust), while maintaining computational efficiency suitable for real-world agricultural applications?”

This study aims to address these gaps and answer the mentioned research question by developing a hybrid model combining EfficientNetB0 and SVM, tailored for multiple crops and diseases. Our objectives are to enhance accuracy, improve computational efficiency, and create a model that can be applied to diverse agricultural contexts in Bangladesh.

## 2. Methodology

**Figure 1:**
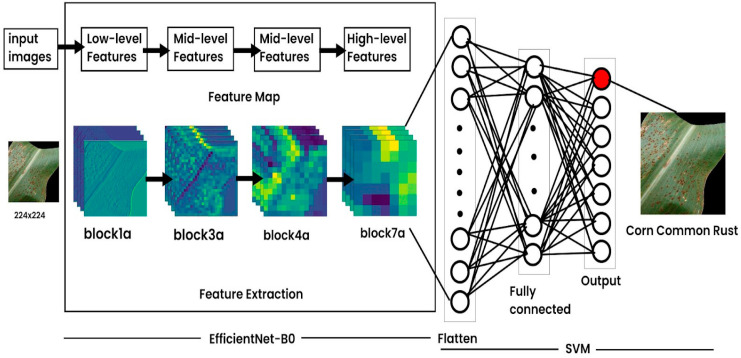
Hybrid model architecture.

This work introduces a hybrid model to predict crop diseases for three types of crops: rice, potato and corn. A pre-processed input image is fed into EfficientNet for feature extraction. Figure 1 illustrates the model architecture where it is shown how the features are being extracted in the blocks of EfficientNet. EfficientNet B0 consists of multiple layers that progressively extract features from input images. These layers can be broadly categorized into low-level, mid-level, and high-level layers based on the abstraction level of the features they capture. The low-level layers usually consist of the initial convolutional and pooling layers. These layers operate directly on the raw pixel values of the input image, convolving over small regions of the image to detect simple patterns like edges and gradients. The mid-level layers are often found in the middle of the network architecture. They learn to detect more sophisticated patterns and textures in the input images, such as object parts, shapes, and contours. High-level layers are located towards the end of the network. These layers find object categories or scene types. The high-level layers integrate information from lower-level and mid-level layers. The extracted features are then passed through the fully connected layers after flattening. Then, the SVM is used as a classifier for disease prediction.

### 2.1. Dataset Preparation

#### 2.1.1. Data Preprocessing

The datasets used in this study include open-source images from Kaggle [24], as well as 1334 images collected by the authors from different fields across different parts of Bangladesh. However, the data we collected was not sufficient enough, so we decided to collect data from open-source datasets as well. There are 7000 images in our dataset after performing data preprocessing and augmentation. The dataset was divided into training and testing, with 80% as train dataset and 20% as test dataset. There were 7 classes, Corn Common Rust, Corn Healthy, Potato Healthy, Potato Late Blight, Rice Brown Spot, Rice Healthy, and Invalid Images. Through data preprocessing, we ensured the quality, diversity, and reliability of our dataset. The preprocessing steps outlined below were executed to optimize the images for subsequent model training and evaluation.

#### 2.1.2. Image Resizing

We prioritized images that exhibited clear visual symptoms of crop diseases, ensuring the dataset’s relevance and utility for our research objectives. Additionally, we included images of healthy plants so that our model could classify healthy and affected crops. To deal with inconsistent image resolution, we have performed image resizing. The original image size was 256 × 256 pixels, which was then resized for model training. The image size was set to 224 × 224 to ensure all the fine details were captured during feature extraction.

#### 2.1.3. Normalization

Normalization is a process of translating data into the range [0, 1]. It includes scaling and transforming numerical features in a dataset to a standard scale. It is mostly performed through min-max scaling and standardization scaling. Following resizing, we normalized the pixel values within each image to a standardized range of [0, 1]. By dividing the pixel values by 255, the maximum intensity value, we effectively scaled the pixel values to a common range, decreasing the impact of lighting variations and enhancing model convergence during training.

#### 2.1.4. Data Augmentation

To enhance the diversity of our dataset and improve model generalization, we applied a range of augmentation techniques, including rotation, horizontal and vertical flipping, height and width shift range, and zooming. The ImageDataGenerator class from the Keras library was used for augmentation during model training. We used 5666 original images and applied augmentation techniques, resulting in a total of 7000 images for training.

#### 2.1.5. Train-Test Split

**Figure 2:**
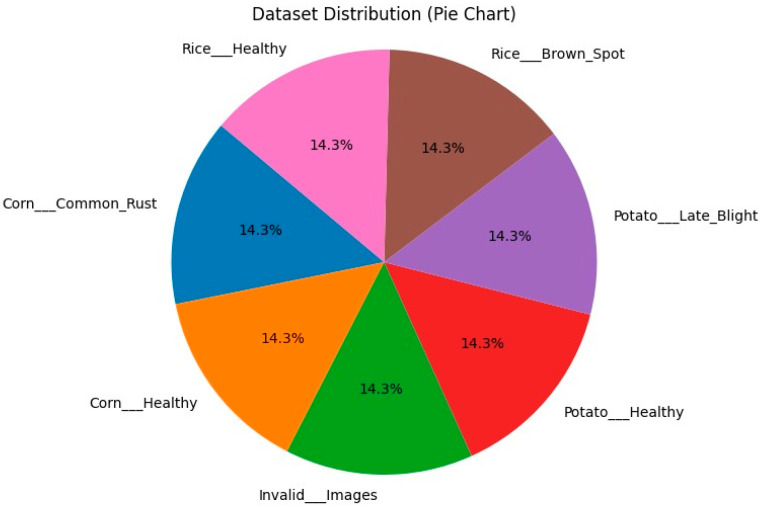
Dataset distribution.

**Figure 3:**
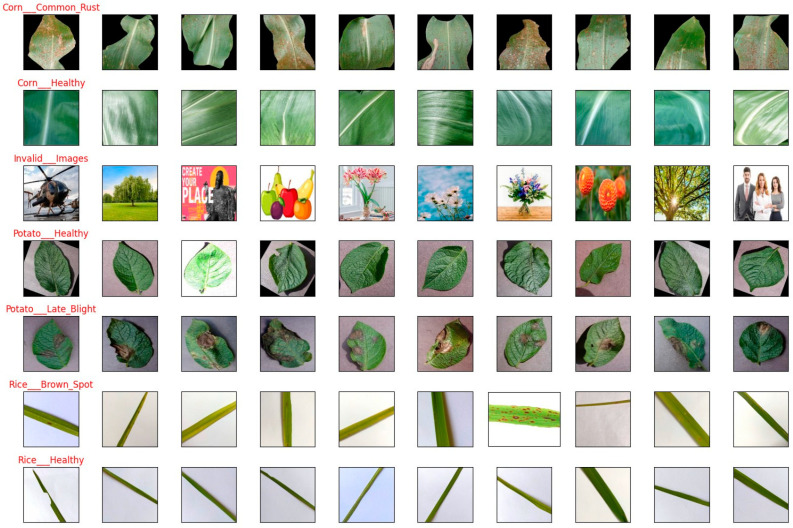
Sample from the dataset.

The preprocessed dataset was divided into training and testing subsets. The subsets had an 80:20 split ratio: 80% of the data was allocated for model training, while the remaining 20% was reserved for testing. There were 5600 images in the training dataset and 1400 in the testing dataset. This was done to evaluate the model’s performance on unseen data while preserving a substantial training dataset for effective model learning. Figure 2 represents the distribution of the final dataset among various classes. Figure 3 shows some of the sample images from the dataset. It is visible that the dataset is balanced as all classes have the same number of samples.

### 2.2. Model Selection

After data pre-processing, an extensive literature study was carried out again. A thorough exploration of various model architectures was conducted to decide on the best approach to create a hybrid model. Starting with a fundamental convolutional neural network (CNN), we proceeded to explore more advanced models such as VGG16, ResNet50, EfficientNetB0, and other CNN models for feature extraction. Then, we decided to choose the most appropriate classification models for our disease detection project. The models we explored and compared with were CNN, VGG16, ResNet50, Xception, Autoencoders, Inception v3, and Efficient B0.

#### 2.2.1. EfficientNetB0 for Feature Extraction

We incorporated EfficientNetB0 for feature extraction which is renowned for its efficiency and scalability in handling image data. EfficientNet’s advanced architecture enabled us to extract informative features from the preprocessed crop images. It helped capture relevant patterns and characteristics from crop images [25]. Capturing intricate patterns is crucial for accurate disease identification. Through EfficientNetB0, we enhanced the discriminative power of our model. This contributed to improving the performance in distinguishing between healthy plants and those affected by various diseases.

#### 2.2.2. SVM for Classification

After feature extraction using EfficientNet B0, a support vector machine (SVM) was integrated for crop disease classification. SVMs excel at establishing intricate decision boundaries based on the extracted features. This synergy between EfficientNet’s feature representation and SVM’s classification capabilities allowed for accurate disease identification within the crop imagery. By combining these techniques, we leveraged both strengths, particularly SVM’s ability to handle high-dimensional data effectively, ultimately leading to a more robust and precise disease detection system [26].

#### 2.2.3. Hybrid Model

Our investigation, through experimentation and performance evaluation, revealed that the combination of EfficientNetB0 for feature extraction and SVM for classification achieved promising results in crop disease detection [27]. This pairing exhibited superior accuracy compared to alternative models. The selection of EfficientNetB0 and SVM stemmed from their complementary strengths, creating a model architecture well-suited to the intricate challenges of crop disease identification.

#### 2.2.4. EfficientNet Architecture

EfficientNet’s innovative approach to scaling, called compound scaling, utilizes a coefficient (φ) to proportionally adjust the network’s width, depth, and resolution. This ensures balanced scaling across these dimensions, achieving an optimal trade-off between model complexity and performance, as referenced in [28,29,30]. The specific scaling factors for width, depth, and resolution are determined through empirical evaluations (Equations (1)–(3)). These equations represent width (w), depth (d), and resolution (r).

Width: w = β·φ,(1)

Depth: d = α·φ,(2)

Resolution: r = γ·φ,(3)

The constants α, β, and γ are established through experimentation to achieve the most effective balance for a particular task.

**Figure 4:**
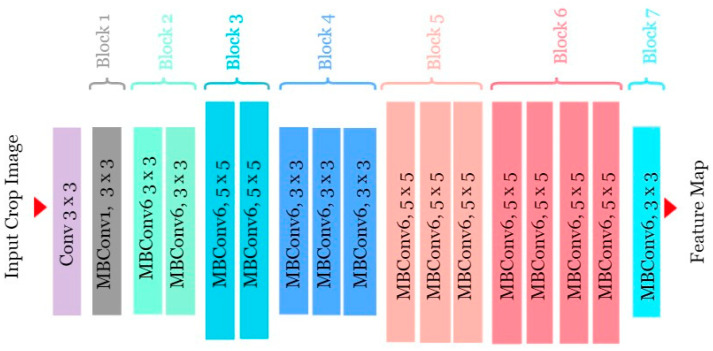
EfficientNetB0 model architecture.

The input layer receives the input data [31]. EfficientNet B0 contains a series of convolutional layers that perform feature extraction from the input images. These convolutional layers use filters/kernels to convolve over the input images, extracting features at different spatial scales. Batch normalization layers are often used afterwards to normalize the activations. The activation layers introduce non-linearities into the network, enabling it to learn complex patterns and relationships in the data [29]. At the end of the convolutional layers, EfficientNet includes a global average pooling layer. This layer accumulates feature maps by taking the average of each feature map. Following that, there may be one or more fully connected layers. Finally, the output layer produces the final predictions of the model. For classification tasks, this layer typically consists of ReLU or softmax activation units corresponding to the number of classes in the dataset. Figure 4 represents the architecture of EfficientNetB0. Table 2 describes the layers in detail for the model.

#### 2.2.5. Transfer Learning

**Figure 5:**
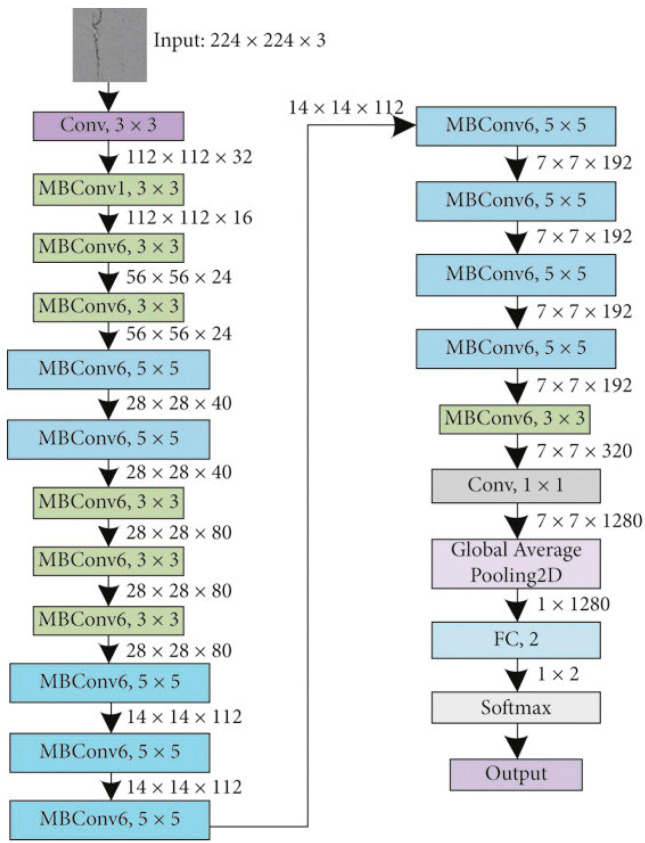
Hybrid model structure diagram.

Through transfer learning, we fine-tuned a pre-trained Efficient Net B0 model using our crop disease dataset. By initializing the model with weights pre-trained on the Image Net dataset, the model efficiently leveraged high-level feature representations learned from a diverse range of images. Fine-tuning involved updating the parameters of the final layers to adapt the model to our specific task while retaining the knowledge gained from Image Net. Mathematically, this can be represented by adjusting the model parameters to minimize the loss function L. Figure 5 illustrates the hybrid model structure.

#### 2.2.6. Feature Extraction Process

In the feature extraction phase, the input images of crops underwent a transformative journey through the layers of the EfficientNetB0 model. This process unfolded as follows:

- Input images: At the outset, our crop disease detection system receives input images depicting various crops, such as corn, rice, or potatoes [32]. These images serve as the raw data input into the EfficientNetB0 model, capturing visual information about the crops’ appearance and condition.
- Propagating through layers: Once the input images are fed into the model, they propagate through the layers of the EfficientNetB0 architecture. The model learns from its errors through backpropagation.
- Extraction of abstract features: As the images traverse through the network, the successive layers extract increasingly abstract and complex features [33,34]. Initially, lower layers may detect simple patterns like edges and textures, while deeper layers capture more sophisticated structures and arrangements specific to crop diseases.
- Aggregation and processing: The extracted features from different layers are aggregated and processed further as they progress through the network [35,36]. Through the process of feature fusion and refinement, the model learns to combine and manipulate the extracted features to enhance their representational power.

#### 2.2.7. Model Training

We set different learning rates during model training. We monitored the model’s performance on a separate validation dataset. The training process was halted if the validation loss did not improve for a certain number of epochs. We adjusted the dropout rate to achieve better results.

We experimented with different batch sizes and epochs to find the optimal configuration for training the model. A batch size of 64 was chosen to balance computational efficiency and model convergence [37,38]. The model was trained for 100 epochs to ensure convergence and capture complex patterns in the data.

## 3. Results and Discussion

This model was experimented on in two stages: training and testing. The hybrid model, combining EfficientNetB0 as a feature extractor with a support vector machine (SVM) classifier, demonstrated better performance for the specified classes—rice, potato, and corn.

**Figure 6:**
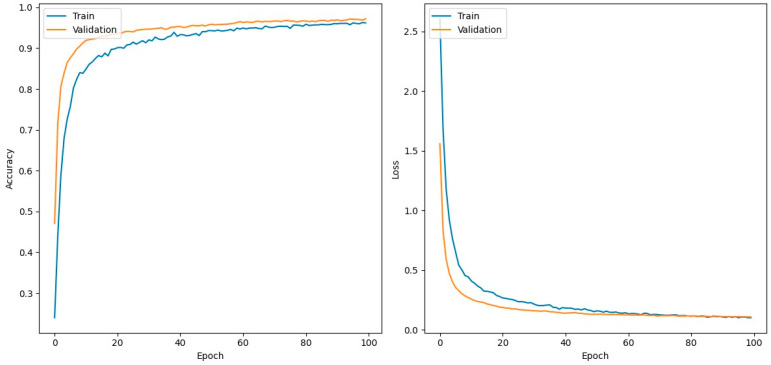
Hybrid plot training & validation accuracy values and validation loss values.

Figure 6 represents the accuracy and loss values of the hybrid model of training and testing. Table 3 and Table 4 describe the classification reports for training and testing respectively where it is observed that the training accuracy is 99.63% and the testing accuracy is 97.29%. The reports also include precision, recall, and F1-score to provide a detailed understanding of the model’s performance for individual classes. These metrics were calculated using a weighted average across classes.

Table 5 illustrates the comparison among various contemporary deep learning models including the proposed hybrid model based on accuracy. The batch size (64), epoch (15), and learning rate were kept the same to conduct a fair comparison. It is observed that our hybrid model achieved the best performance out of all these models with an accuracy of 97.29%. The results indicate that the proposed hybrid model achieved a high accuracy for all three diseases, demonstrating its potential for practical use. However, further validation in real-world conditions is necessary to confirm its effectiveness.

## 4. Conclusions

In conclusion, this study presents a robust framework for crop disease prediction in Bangladesh, a nation where such capabilities are paramount for guaranteeing long-term agricultural productivity and food security. Although we utilized open-source datasets, a significant portion of our data, comprising 23.54%, was collected from agricultural fields in Bangladesh. By strategically integrating deep learning methodologies, specifically EfficientNetB0, with the established classification prowess of support vector machines (SVM), we have developed a hybrid model optimized for disease classification across three vital crops: rice, potato, and corn. This model capitalizes on EfficientNetB0′s proficiency in feature extraction, demonstrably leading to enhanced performance and accuracy. Notably, the proposed hybrid model achieves a commendable accuracy of 97.29%, surpassing the performance of several benchmark models during comparative analysis. This study makes a valuable contribution to the field of plant disease identification by demonstrating the effectiveness of deep learning models across multiple crops. However, further research is needed to enhance the models’ robustness and adaptability to diverse agricultural environments. Future endeavors could involve expanding this approach to encompass a wider range of crops and integrating real-time monitoring systems. Such advancements have the potential to further bolster agricultural sustainability and resilience.

## Figures and Tables

**Table 1:** Literature Analysis.

Model	Traditional ML	Inception	CNN	ResNet	MobileNetV2	VGGNet	YOLO	DenseNet	GoogleNet	EfficientNet
Paper [5]						✓		✓		✓
Paper [8]			✓							
Paper [12]		✓		✓		✓		✓		
Paper [13]						✓				
Paper [14]	✓ (RF)	✓			✓	✓				
Paper [15]			✓							
Paper [16]			✓							
Paper [17]				✓						
Paper [18]		✓		✓		✓				✓
Paper [19]			✓							
Paper [20]										✓
Paper [21]				✓	✓	✓		✓	✓	
Paper [22]										✓
Paper [23]										✓

**Table 2:** EfficientNetB0 model table.

Stage i	Operator	Resolution	No. of Channels	No. of Layers
1	Conv3×3	112 × 112	32	1
2	MBConv1, k3×3	112 × 112	16	1
3	MBConv6, k3×3	112 × 112	24	2
4	MBConv6, k5×5	56 × 56	40	2
5	MBConv6, k3×3	28 × 28	80	3
6	MBConv6, k5×5	14 × 14	112	3
7	MBConv6, k5×5	7 × 7	182	4
8	MBConv6, k3×3	7 × 7	320	1
9	Conv1×1 & Pooling & FC	7 × 7	1280	1

**Table 3:** Hybrid classification report (train).

	Precision	Recall	F1-Score	Support
Corn Common Rust	1	1	1	800
Corn Healthy	0.996264	1	0.9981285	800
Invalid Images	1	1	1	800
Potato Healthy	1	1	1	800
Potato Late Blight	1	1	1	800
Rice Brown Spot	1	0.9825	0.9868173	800
Rice Healthy	0.9911728	0.99125	0.9887781	800
Accuracy			0.99625	5600
Macro Average	0.9962507	0.99625	0.9962463	5600
Weighted Average	0.9962507	0.99625	0.9962463	5600

**Table 4:** Hybrid classification report (test).

	Precision	Recall	F1-Score	Support
Corn Common_Rust	1	1	1	200
Corn Healthy	0.990099	1	0.9950249	200
Invalid Images	1	1	0.9974937	200
Potato Healthy	0.9848485	1	0.9798995	200
Potato Late Blight	0.9752475	1	0.9800995	200
Rice Brown Spot	0.952381	0.9825	0.9254499	200
Rice Healthy	0.9095238	0.99125	0.9317073	200
Accuracy			0.9728571	1400
Macro Average	0.9731571	0.9728521	0.9728107	1400
Weighted Average	0.9731571	0.9728521	0.9728107	1400

**Table 5:** Comparative analysis of ResNet50, VGG16, CNN, EfficientNetB0, Xception, mobilenetV2, Autoencoders, Inception V3, Hybrid.

Model	Input Shape	Train Accuracy	Test Accuracy
CNN	128, 128, 3	91.20%	86.57%
VGG16	128, 128, 3	85.98%	83.29%
ResNet50	128, 128, 3	71.75%	68.79%
Xceptiion	128, 128, 3	95.05%	94.07%
Mobilenet V2	128, 128, 3	92.00%	90.71%
Autoencoders	128, 128, 3	87.98%	87.90%
Inception v3	128, 128, 3	85.21%	94.14%
EfficientnetB0	128, 128, 3	98.025	96.14%
Hybrid	224, 224, 3	99.63%	97.29%

## Data Availability

All the data used in this study can be obtained from the corresponding author upon a reasonable request.
